# LiPo batteries dataset: Capacity, electrochemical impedance spectra, and fit of equivalent circuit model at various states-of-charge and states-of-health

**DOI:** 10.1016/j.dib.2023.109561

**Published:** 2023-09-12

**Authors:** Matteo Galeotti, Lucio Cinà, Corrado Giammanco, Aldo Di Carlo, Francesco Santoni, Alessio De Angelis, Antonio Moschitta, Paolo Carbone

**Affiliations:** a Department of Electronic Engineering, University of Rome “Tor Vergata”, *Via* del Politecnico, Rome 00133, Italy; b Cicci Research srl., *Via* Giordania 227, Grosseto 58100, Italy; c ISM-CNR, Istituto di Struttura della Materia, Consiglio Nazionale delle Ricerche, *Via* Fosso del Cavaliere 100, Rome 00133, Italy; d Department of Engineering, University of Perugia, *Via* Goffredo Duranti 93, Perugia 06125, Italy

**Keywords:** Electrochemistry, Spectroscopy, Battery charging, Lithium battery, Impedance, Lithium battery state-of-charge, Lithium batteries state-of-health

## Abstract

This dataset contains experimental data of capacity and electrochemical impedance of five Lithium Polymer (LiPo) batteries (model LP-503562-IS-3 manufactured by BAK Technology). All batteries have been subjected to hundreds of charge-discharge cycles to obtain their characteristics at different states-of-health. Capacities have been measured under both standard and stress conditions. At fixed intervals (45 cycles in most cases) batteries have been subjected to partial discharge cycles to measure impedance spectra at different values of the state-of-charge. Impedance spectra have been fitted by using an equivalent circuit model; estimated circuit parameters are included in the dataset.

Specifications Table

**Table:** 

Subject	Energy Engineering and Power Technology
Specific subject area	Capacity measurements and electrochemical impedance spectroscopy at different states-of-charge and states-of-health on 3.7 V LiPo batteries with nominal capacity 1.1 Ah.
Type of data	Table
How the data were acquired	3.7 V LiPo batteries (model LP-503562-IS-3 manufactured by BAK Technology, nominal capacity 1.1 Ah) have been cycled by means of a source measure unit (SMU). Standard charge and discharge have been performed using 1 A current. Stress discharge has been performed using 3 A current. Electrochemical impedance spectroscopy (EIS) has been performed by means of a galvanostat. Single sine signals have been used as excitation signal, selecting 45 values of frequency, ranging from 0.2 to 5000 Hz. The current signal amplitude was 40 mA. Parameter fitting of an equivalent circuit model (ECM) has been performed by means of non-linear least-square method with suitable initial conditions.
Data format	Raw Analyzed
Description of data collection	During measurement procedures, batteries were kept at constant temperature of 25 °C thanks to a Peltier cell. After discharge, EIS data were acquired after a relaxation time of 5 min in order to stabilize the open circuit voltage (OCV) and the temperature. Charge-discharge cycles have been repeated until the capacity C dropped below 70 % of the nominal capacity $C_{N}$
Data source location	· Institution: Department of Electronic Engineering, University of Rome “Tor Vergata” · City: Rome 00133 · Country: Italy · Latitude and longitude: 41.855759°, 12.624262°
Data accessibility	Repository name: LiPo Battery LP-503562-IS-3 EIS, Capacity, ECM Data Data identification number: 10.17632/stcppt2r68.1 Direct URL to data: https://data.mendeley.com/datasets/stcppt2r68/1
Related research article	M. Galeotti, L. Cinà, C. Giammanco, S. Cordiner, A. Di Carlo, Performance analysis and SOH (state of health) evaluation of lithium polymer batteries through electrochemical impedance spectroscopy. Energy, 89, 678-686, 2015

## 1 Value of the Data

• The dataset [1] comprises capacity and EIS measurements on commonly used LiPo batteries at different states-of-charge (SOC) and states-of-health (SOH). The dataset includes batches of measurements taken from five different cells of the same manufacturer and model (LP-503562-IS-3 by BAK Technology).
• These data allow the analysis of frequency-domain characteristics of batteries and can serve as a valuable resource for developing estimation methods and training machine learning models with the objective of designing diagnostic tools. In particular, EIS data can be exploited to extract information on the SOC, SOH, and remaining useful life (RUL), as well as for fault diagnosis [2,3].
• The dataset can be of interest to researchers in the field of electrochemistry, and to electronic engineers, particularly for the design of battery management systems (BMS) [4,5].
• The growing body of literature exploring data-driven methods for battery applications makes datasets such as [1] an important resource for research on battery applications. Extensive acquisition of battery data can be expensive and is generally time-consuming. The availability of public datasets is therefore valuable to the research community. Dataset [1] substantially adds to this line [6,7].

## 2 Objective

This dataset was originally acquired for the purpose of studying the aging effects of LiPo batteries, resulting in a research paper where a machine learning technique based on the Dempster–Shafer theory was applied to estimate the SOH [2]. The publication of this dataset contributes to the repeatability of results, and to the validation of multiple techniques for battery analysis and parameter estimation.

## 3 Data Description

Experimental data are stored into folders /Lipo_*i,* where i=1,2,3,4,5 identifies each of the five batteries that have been characterized. Equivalent circuit model fitted parameters are stored into folders /Lipo_*i*_fit. Each battery has undergone hundreds of complete charge and discharge cycles. From now on, an x in file names identifies cycle number. The point “.” in folder names identifies the folder /Lipo_*i*. As detailed in the experimental section, measurements are divided into three stages (0, 1 and 2), corresponding to capacity data, EIS data, and stress discharge data, respectively.

### 3.1 Capacity Data (stage 0)

**Fig. 1:**
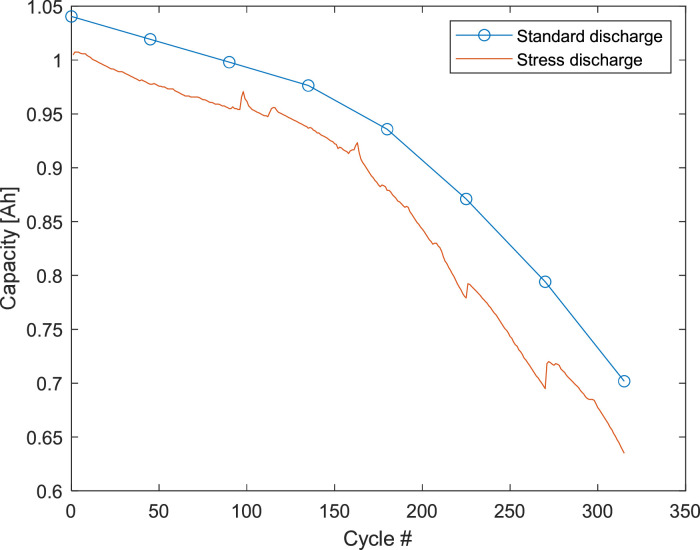
Battery capacity versus cycle number measured during standard discharge (stage 0) and stress discharge (stage 2) of LiPo 4. The same data are shown in Fig. 4 of Ref. [2].

**Fig. 2:**
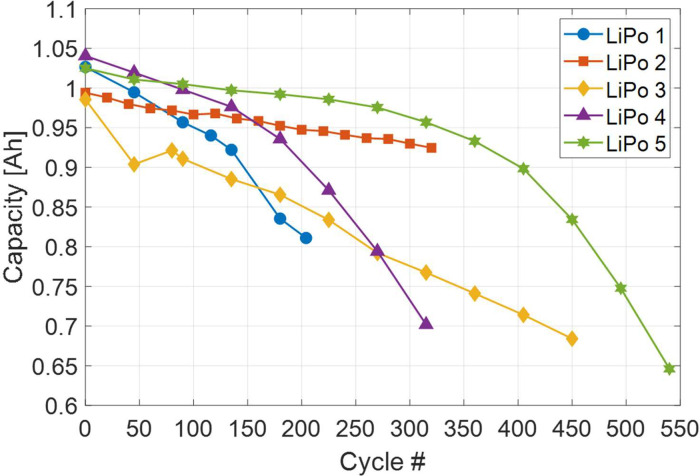
Battery capacity versus cycle number measured during standard discharge for LiPo 1–5.

**Fig. 3:**
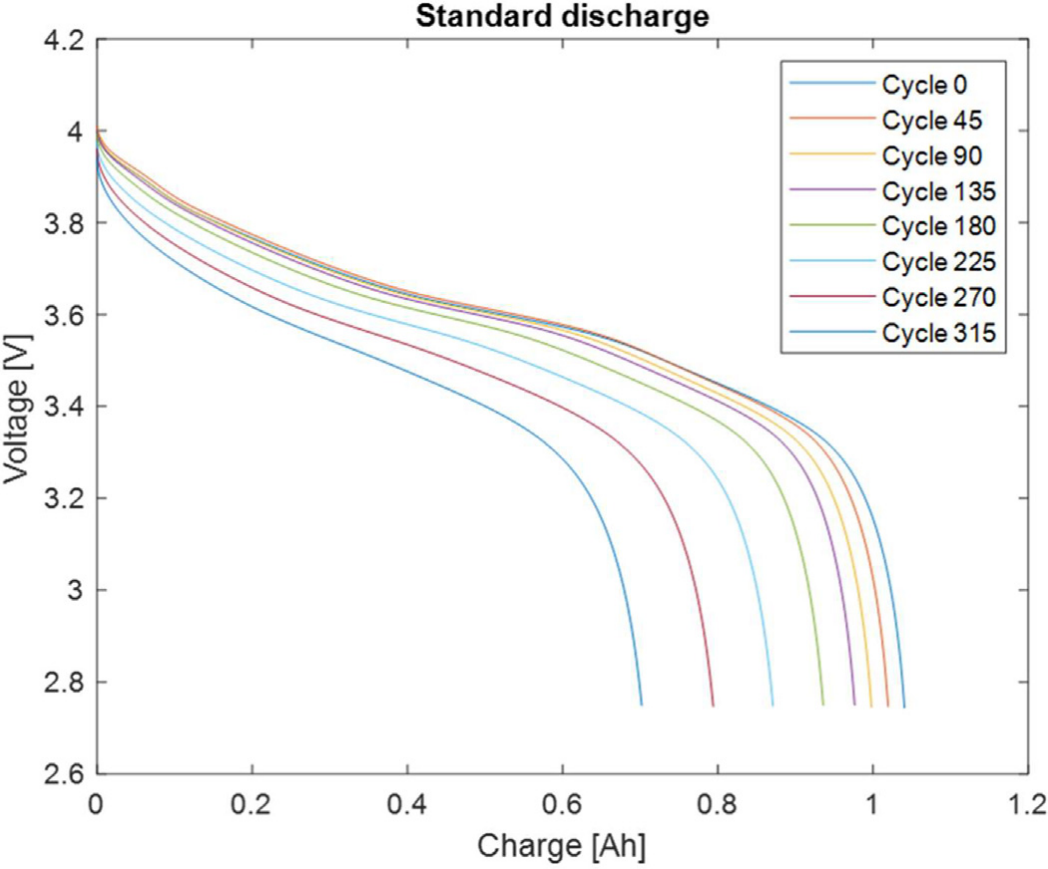
Discharge curves of LiPo 4 for various cycles. The same data are shown in Fig. 4 of Ref. [2].

Capacity measurements involved a complete charge-discharge-charge process during which current-voltage characteristics were measured, resulting in three different sets of comma-separated values (csv) data files (Table 1). Discharge curves have been obtained by integrating current over time (Table 2). Capacity has been estimated from the total extracted charge (see experimental section) and has been reported versus cycle number (Table 3). Data examples are shown in Fig. 1, Fig. 2. In Fig. 1, the capacity versus cycle number for LiPo 4 is reported for standard and stress discharge. Capacity versus cycle number of all batteries for standard discharge is reported in Fig. 2. In Fig. 3, discharge curves for various cycles of LiPo 5 are reported.

**Table 1:** Capacity data (stage 0): current-voltage characteristics of the battery during charge-discharge-charge process.

Folder	Files	Columns		
./EIS_Charge_discharge/	0_*x*_0_stdcharge.csv	Time [s]	Current [A]	Voltage [V]
	0_*x*_1_stddischarge.csv			
	0_*x*_2_stdcharge.csv

**Table 2:** Capacity data (stage 0): discharge curve.

Folder	Files	Columns	
./Discharge_curve/	*x*_Discharge_std.csv	Extracted charge [Ah]	Voltage [V]

**Table 3:** Capacity data (stage 0): capacity of the battery versus cycle number.

Folder	File	Columns	
./Capacity/	Capacity_std.csv	Cycle number	Capacity [Ah]

### 3.2 EIS Data (stage 1)

**Fig. 4:**
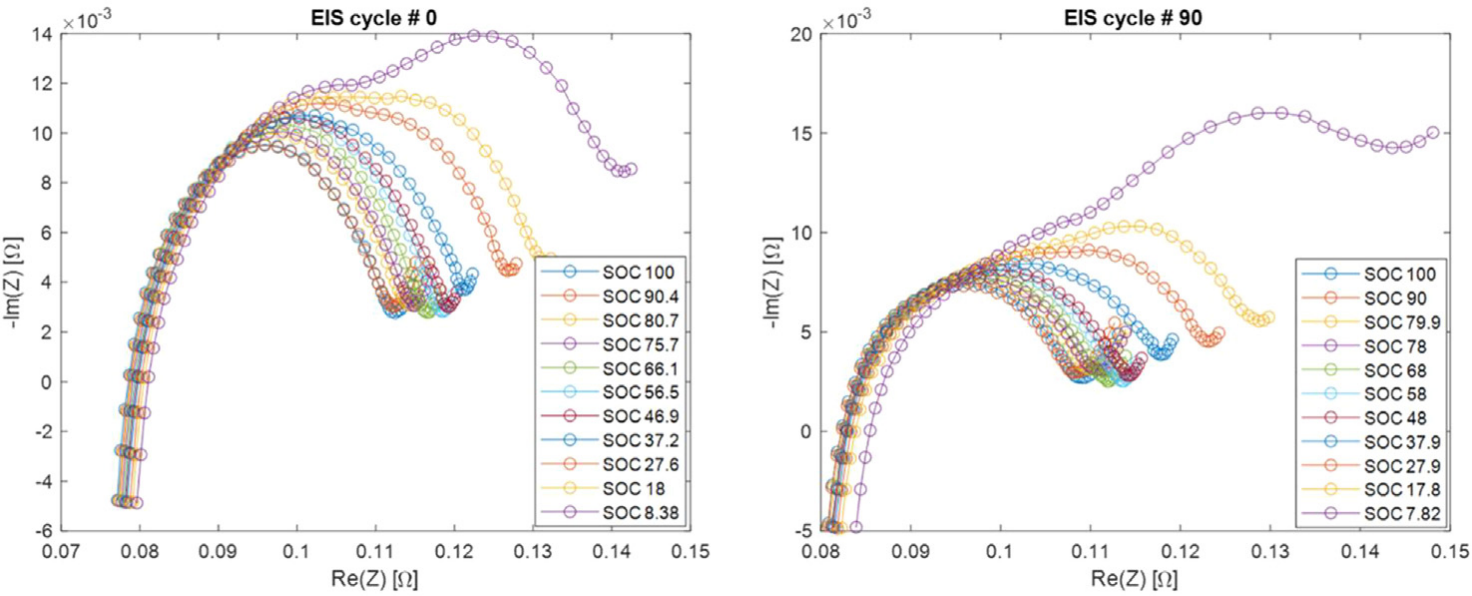
EIS spectra of LiPo 4 at various SOC values at two different SOH (cycle 0 and 90). Part of these data is shown in Figs. 5 and 6 of Ref. [2].

**Fig. 5:**
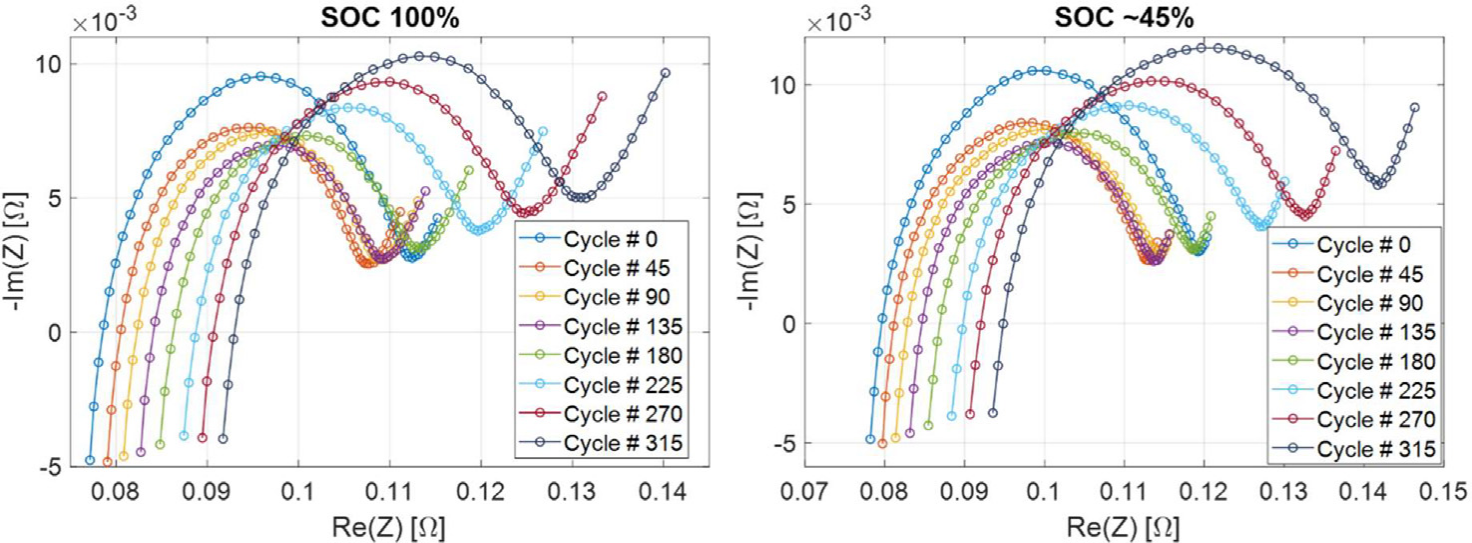
EIS spectra of LiPo 4 at various SOH values at two different SOC (100 % and about 45 %). Part of these data is shown in Figs. 5 and 6 of Ref. [2].

Electrochemical impedance spectra have been measured at different SOC values obtained by partial discharge (see experimental section). SOC is value between 1 (fully charged) and 0 (fully discharged). The complex impedance Z(f) has been measured, where f is the exciting current signal frequency. Real and imaginary part of Z(f) are stored separately. SOC values are reported in Table 4; cycle numbers during which EIS has been performed are reported in Table 5; current-voltage characteristics during partial discharge are reported in Table 6; EIS spectra are reported in Table 7. In order to provide some examples, Nyquist plots of EIS spectra of LiPo 4 at various SOC values and at two different SOH are reported in Fig. 4, while EIS spectra at various SOH values and at two different SOC are reported in Fig. 5.

**Table 4:** EIS data (stage 1): SOC values.

Folder	File	Columns
./EIS_Charge_discharge/EIS_*x*/	SOC.csv	SOC value

**Table 5:** EIS data (stage 1): Cycle numbers during which EIS is performed.

Folder	File	Columns
./EIS_Charge_discharge/	EIS_cycles.csv	Cycle number during which EIS is performed

**Table 6:** EIS data (stage 1): current-voltage characteristics of the battery during partial discharge. In file names, y is an index identifying different SOC values.

Folder	Files	Columns		
./EIS_Charge_discharge/	1_*x*_*y*_pardisch.csv	Time [s]	Current [A]	Voltage [V]

**Table 7:** EIS data (stage 1): EIS spectra at various SOC values.

Folder	Files	Columns		
./EIS_Charge_discharge/EIS_*x*/	*y*_EIS.csv	Frequency [Hz]	Ne(Z) [Ω]	ℑm(Z) [Ω]

### 3.3 Stress Discharge Data (stage 2)

Stress discharge cycles have been performed to accelerate the ageing of batteries. As in stage 0, current-voltage characteristics were measured, resulting in two different sets of files (Table 8). Discharge curves have been obtained by integrating current over time (Table 9). Capacity has been estimated from the total extracted charge and has been reported versus cycle number (Table 10). In Fig. 1, the capacity versus cycle number for LiPo 4 is reported as measured during stress discharge.

**Table 8:** Stress discharge data (stage 3): current-voltage characteristics of the battery during charge-discharge-charge process.

Folder	Files	Columns		
./EIS_Charge_discharge/	2_*x*_stresscharge.csv	Time [s]	Current [A]	Voltage [V]
	2_*x*_stressdischarge.csv

**Table 9:** Stress discharge data (stage 3): discharge curve.

Folder	Files	Columns	
./Discharge_curve/	*x*_Discharge_stress.csv	Extracted charge [Ah]	Voltage [V]

**Table 10:** Stress discharge data (stage 0): capacity of the battery versus cycle number.

Folder	File	Columns	
./Capacity/	Capacity_stress.csv	Cycle number	Capacity [Ah]

### 3.4 Equivalent Circuit Model

**Fig. 6:**
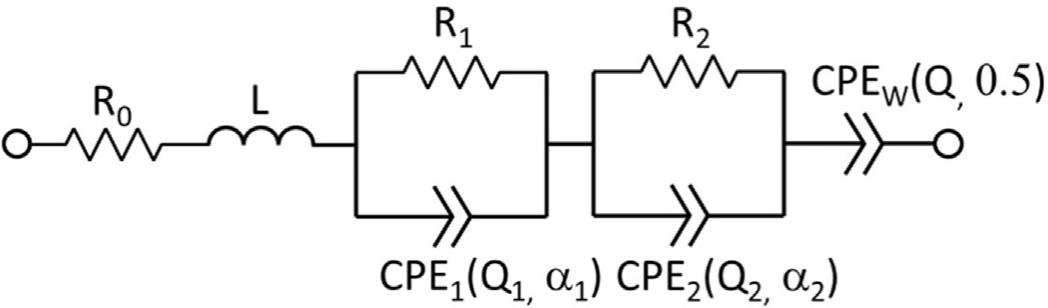
Equivalent circuit model used to fit EIS spectra.

Complex impedance spectra are fitted using the equivalent circuit model (ECM) shown in Fig. 6. The impedance of the constant phase element is $Z_{\text{CPE}}\left(s\right)=\frac{1}{Qs^{\alpha }}$, where s=iω (ω being the angular frequency), α is a dimension-less exponent and Q is a generalized capacitance whose units are $s^{\alpha }\Omega^{-1}$. The CPE with fixed α=0.5 is called Warburg element. The parallel of CPE and resistor is called Randles circuit: $Z_{\text{Randles}}\left(s\right)=Z_{\text{CPE}}\left(s\right)∥R=\frac{R}{1+RQs^{\alpha }}$. L is an inductor. Thus, the total impedance is:

(1) $$Z_{\text{CPE}}\left(s\right)=R_{0}+\frac{R_{1}}{1+R_{1}Q_{1}s^{\alpha_{1}}}+\frac{R_{2}}{1+R_{2}Q_{2}s^{\alpha_{2}}}+\frac{1}{Qs^{0.5}}+sL$$

Hence there are nine parameters $R_{0},\;R_{1},\;Q_{1},\;\alpha_{1},\;R_{2},\;Q_{2},\;\alpha_{2},\;Q,\;L$ that have been fitted to the experimental EIS spectra.

**Fig. 7:**
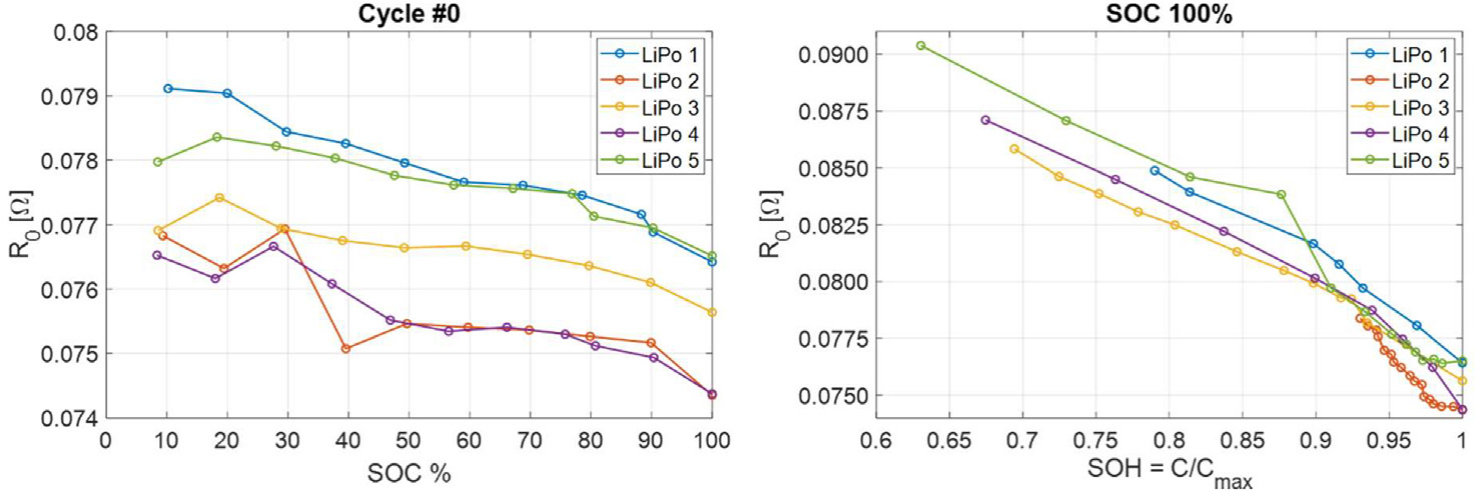
Parameter R0 plotted vs SOC (at Cycle 0) and vs SOH (at SOC 100 %). Since each battery has a different lifetime as measured in number of cycles, on the right plot SOH has been defined as the ratio of actual capacity and maximum capacity of each battery, thus obtaining a consistent parameter to compare different batteries on the same plot.

Fitted parameters are stored into folders /Lipo_*i*_fit. Into each LiPo folder, there are two kinds of files model_fit_cycle_*x*.csv and model_fit_err_cycle_*x*.csv. In the first one, the parameter values are stored; in the second, their standard deviation is stored. The columns of both files are organized as in Table 11. As an example, the variation of the parameter $R_{0}$ vs SOC and SOH is reported in Fig. 7.

**Table 11:** ECM parameters order in model_fit_cycle_*x*.csv and model_fit_err_cycle_*x*.

SOC	$R_{0}\;\left[\Omega \right]$	$R_{1}\;\left[\Omega \right]$	$Q_{1}\;\left[s^{\alpha_{1}}\Omega^{-1}\right]$	$\alpha_{1}$	$R_{2}\;\left[\Omega \right]$	$Q_{2}\;\left[s^{\alpha_{2}}\Omega^{-1}\right]$	$\alpha_{2}$	$Q_{1}\;\left[s^{0.5}\Omega^{-1}\right]$	L[H]

## 4 Experimental Design, Materials and Methods

**Fig. 8:**
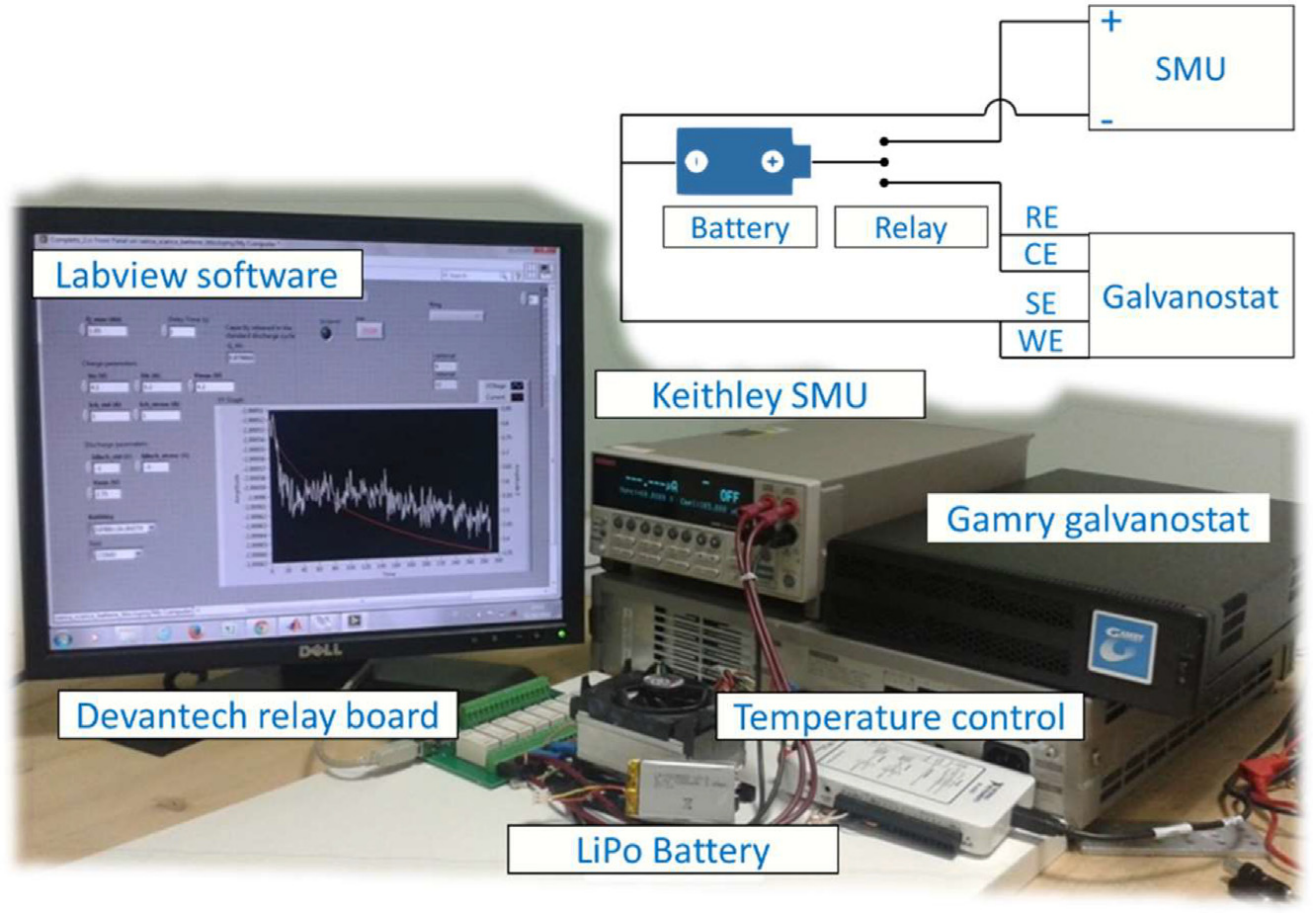
A picture of the experimental setup. A scheme of battery and instruments connections is shown on the top. RE: reference electrode; CE: counter electrode; SE: sensing electrode; WE: working electrode.

LiPo batteries have been cycled by means of a source measure unit (SMU) Keithley 2420. Electrochemical impedance spectroscopy (EIS) has been performed by means of a galvanostat Gamry Instruments Series G 300. A relay board Devantech RLY816 was used to commute battery connection between SMU and galvanostat. The experimental setup was controlled through LabVIEW. During measurement procedures, batteries were kept at constant temperature of 25 °C thanks to a Peltier cell. In Fig. 8, a picture of the experimetal setup is shown with a scheme of battery and instruments connections. The voltage range of the Keithley SMU was set to 20 V, with a resolution of 500 μV, while current range was set to 3 A, with a resolution of 50 μA. The voltage range of the Gamry galvanostat was set to ±30 V, with a resolution of ±1 mV, while current range was set to 300 mA, with a resolution of 900 μA.

**Fig. 9:**
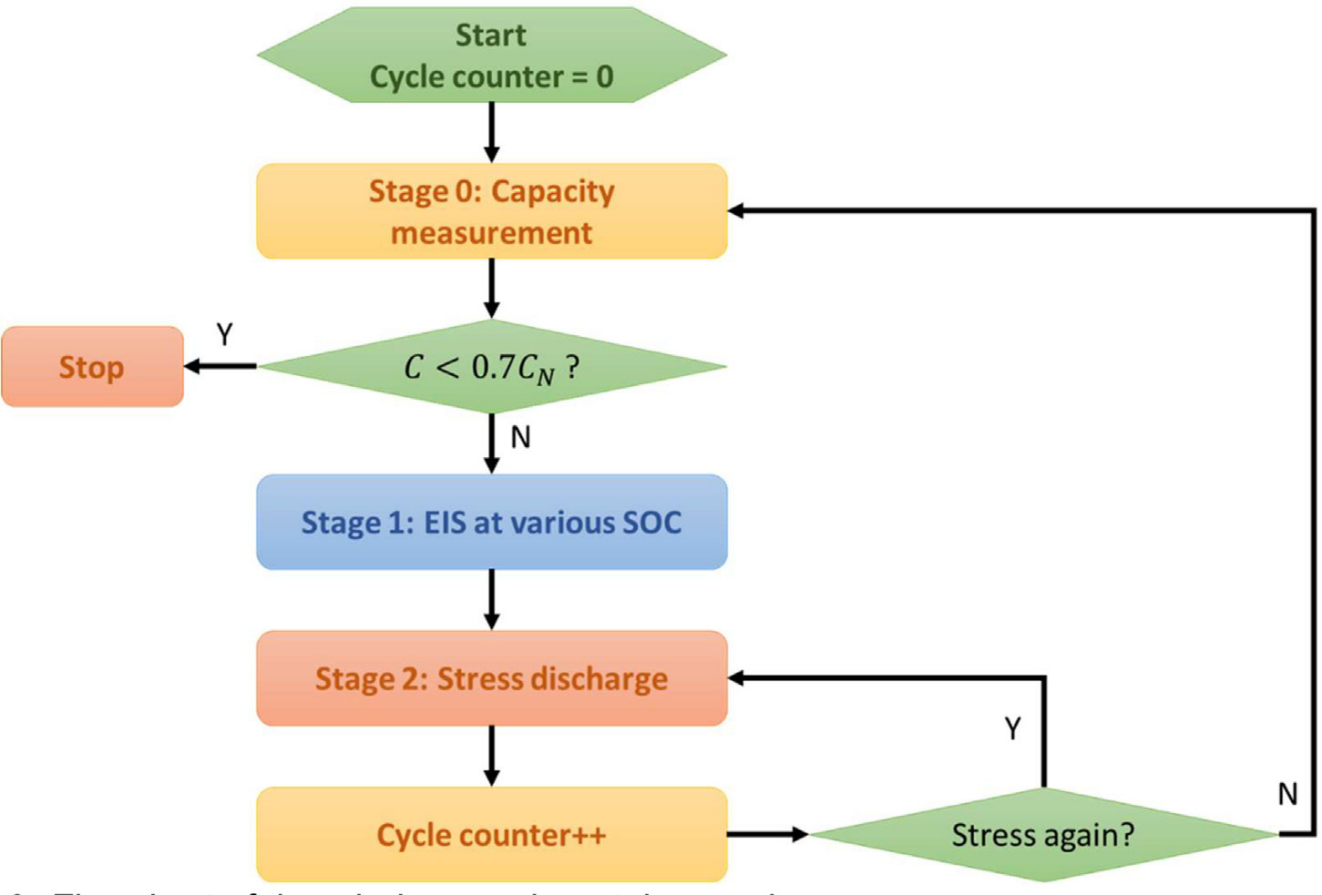
Flowchart of the whole experimental procedure.

**Fig. 10:**
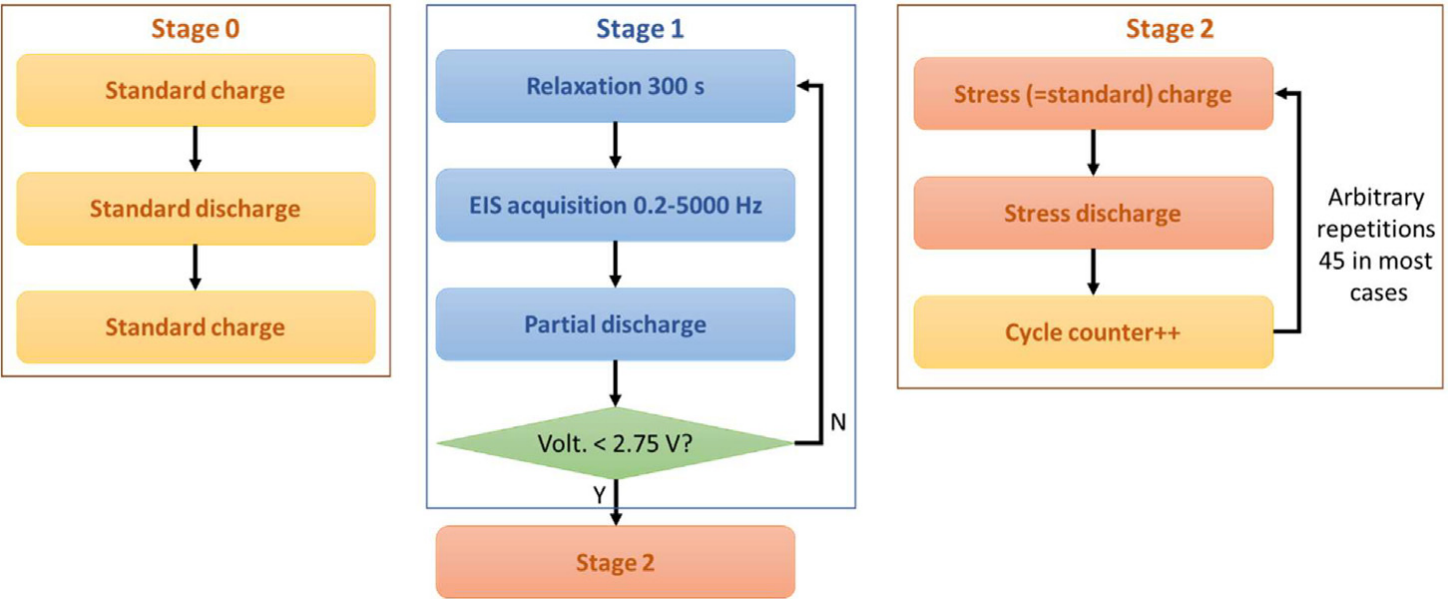
Detailed flowcharts of the whole experimental procedure.

Let us call C and $C_{N}$ the measured and nominal capacity respectively. The whole experimental procedure can be outlined by the flowchart in Fig. 9, which can be split into three different stages detailed in Fig. 10. The procedure is repeated until C drops below $0.7C_{N}$, a threshold chosen to identify the end of battery life.

### 4.1 Stage 0

At stage 0, the battery is charged using the *standard charge* protocol. Then, in order to measure the capacity C, it is discharged using *standard discharge* protocol. Finally, the battery is charged again before moving to stage 1.

*Standard charge:*

(1) Constant current 1 A is applied until voltage is 4.2 V.
(2) Constant voltage 4.2 V is applied until current drops to 0.2 A.

*Standard discharge*:

(1) Constant current 1 A is applied until voltage drops to 2.75 V.

Capacity is estimated by integrating current over discharge time:

(2) $$C=\int_{0}^{t_{\text{discharge}}}I\left(t\right)dt$$

*Stage 1*

At stage 1, different SOC values are obtained by applying the *partial discharge* protocol. At each SOC, EIS is performed by means of single sine current signals with frequencies ranging from 0.2 to 5000 Hz. Current signal amplitude is 40 mA. When voltage drops below 2.75, stage 1 is over.

*Partial discharge:*

(1) Constant current 1 A is applied for 360 s.
(2) A relaxation time of 300 s is allowed to stabilize OCV and temperature.

SOC values are estimated by integrating current over time of partial discharge:

(3) $$SOC=1-\frac{1}{C}\int_{0}^{t_{\text{part}.\;\text{disch}.}}I\left(t\right)dt$$

SOC is estimated by using the capacity value C measured at stage 0, i.e., C is updated as the battery ages.

*Stage 2*

At stage 2, stress charge-discharge cycles are arbitrarily repeated (45 repetitions in most cases). Battery capacity during stress-discharge is estimated using Eq. (2). At the end of stage 2, the procedure restarts at stage 0.

*Stress charge* ≡ *standard charge:*

*Stress discharge:*

(1) Constant current 3 A is applied until voltage drops to 2.75 V.
(2) Cycle counter is increased. The count of cycles defines the SOH.

### 4.2 Equivalent Circuit Model

ECM parameters have been fitted by solving a non-linear least square problem minimizing the cost function ϕ:

(4) $$\phi \left(\theta \right)=\sum_{n=1}^{N}\left\{{\left[Ne\left(\tilde{Z}\left(s_{n}\right)-Z\left(s_{n},\;\theta \right)\right)\right]}^{2}+{\left[ℑm\left(\tilde{Z}\left(s_{n}\right)-Z\left(s_{n},\;\theta \right)\right)\right]}^{2}\right\},$$

(5) $$\hat{\theta }=\underset{\theta }{\text{arg}\;\text{min}}\phi \left(\theta \right),$$

where, N is the number of observed frequencies, $\tilde{Z}$ is the measured impedance, $Z(s_{n},\;\theta )$ is the impedance model (3), and θ is the vector of model parameters. The non-linear optimization problem has been solved in Matlab using the function lsqnonlin. Let us define the vector $\left(\theta \right)=\;{\left[Z\left(s_{1},\;\theta \right)\cdots Z\left(s_{N},\;\theta \right)\right]}^{T}$ . The standard deviation $\sigma_{\theta }\;$of fitted parameters has been estimated as [8]:

(6) $${\hat{\sigma }}_{\theta }=\sigma \sqrt{\text{diag}{\left(J^{T}J\right)}^{-1}},$$

where, J is the Jacobian matrix $J={\frac{\partial {\left[\begin{array}{cc} NeZ^{T} & ℑmZ^{T} \end{array}\right]}^{T}}{\partial \theta }|}_{\theta =\hat{\theta }}$, and σ is the standard deviation of noise, which is assumed to be zero-mean and uncorrelated. σ has been estimated as:

(7) $$\hat{\sigma }=\sqrt{\frac{\phi \left(\hat{\theta }\right)}{2N-p}},$$

where, p=9 is the number of estimated parameters.

Model (3) is non-injective, indeed the two Randles circuits are interchangeable. The model is thus non-identifiable, unless, following [9], the order of parameters is fixed as $\tau_{1}<\tau_{2}$, where $\tau_{1,2}={\left(R_{1,2}Q_{1,2}\right)}^{\frac{1}{\alpha_{1,2}}}$. Furthermore, in order to avoid non-optimal local minima, initial condition of the non-linear optimization algorithm have been chosen according to the indications in Ref. [10], and a multi-start algorithm, performing random search on a neighborhood of chosen initial values, has been used.

## Ethics Statements

No human subjects or animals were involved in this works. No data was collected from social media platforms.

## CRediT authorship contribution statement

**Matteo Galeotti:** Conceptualization, Methodology, Investigation, Software, Data curation, Writing – original draft, Writing – review & editing. **Lucio Cinà:** Conceptualization, Methodology, Investigation, Writing – review & editing. **Corrado Giammanco:** Conceptualization, Methodology, Investigation, Writing – review & editing. **Aldo Di Carlo:** Conceptualization, Methodology, Writing – review & editing, Supervision. **Francesco Santoni:** Conceptualization, Methodology, Formal analysis, Software, Data curation, Writing – original draft, Writing – review & editing. **Alessio De Angelis:** Conceptualization, Methodology, Software, Writing – review & editing. **Antonio Moschitta:** Conceptualization, Methodology, Resources, Writing – review & editing. **Paolo Carbone:** Conceptualization, Methodology, Writing – review & editing, Supervision.

## Data Availability

LiPo Battery LP-503562-IS-3 EIS, Capacity, ECM Data (Original data) (Mendeley Data).
